# Novel topical dressing for dry socket and comparison of its efficacy with that of Alvogyl®: A randomized controlled clinical trial

**DOI:** 10.4317/medoral.26820

**Published:** 2024-11-25

**Authors:** Khashayar Famili, Mahdi Gholami, Arsalan Shahri

**Affiliations:** 1 Assistant Professor of Oral and Maxillofacial Surgery, Mashhad University of Medical Sciences, Mashhad, Iran; 2 Associate Professor of Oral and Maxillofacial Surgery, Mashhad University of Medical Sciences, Mashhad, Iran; 3 Orcid: 0000-0002-0980-7797. Dentist, Research Assistant, Dental Materials Research Center, Mashhad University of Medical Sciences, Mashhad, Iran

## Abstract

**Background:**

To evaluate the efficacy of a novel topical dressing (composed of triamcinolone, ground Dianthus caryophyllus, eugenol, honey, and Iris germanica) for alveolar osteitis (dry socket) against Alvogyl® (composed of eugenol, butamben, and iodoform).

**Material and Methods:**

In a randomized parallel-armed clinical trial at Mashhad Dental School's Department of Oral and Maxillofacial Surgery, 36 patients with alveolar osteitis were randomly allocated into two groups according to the inclusion criteria (*n*=18), using sealed envelopes: one receiving a novel topical dressing and the other receiving Alvogyl®. Post-treatment pain was assessed using a visual analog scale immediately after the procedure and at 30 and 60 minutes, 24, 48, 72, and 96 hours, and one week later, as well as the frequency of dressing applications and analgesic usage. Data analysis was performed using SPSS version 26.

**Results:**

Analyses were completed on all 36 participants without dropouts. No significant age or gender differences were found between the groups at baseline (*p*=0.370 and *p*=0.502, respectively). The novel dressing group experienced significantly lower pain scores at 30 and 60 minutes post-treatment (*p*<0.001), but higher scores at 24 (*p*=0.029), 48 (*p*=0.001), and 72 (*p*=0.017) hours, and similar pain scores immediately after the procedure and at 96 hours and 1 week (*p*>0.05), compared to the Alvogyl® group. The mean number of analgesics taken (*p*=0.097) and the mean frequency of dressing application (*p*=0.839) were not significantly different between the two groups.

**Conclusions:**

The novel topical dressing demonstrated efficacy comparable to Alvogyl®, with the added benefits of cost-effectiveness and the absence of side effects, suggesting its potential as an alternative treatment for dry socket.

** Key words:**Dry socket, alveolar osteitis, butyl aminobenzoate, eugenol, iodoform, spearmint oil, drug combinations.

## Introduction

Alveolar osteitis, or dry socket, is a common complication following tooth extractions, initially identified by Crawford in 1896 (1). It is characterized by worsening postoperative pain 1 to 3 days after extraction, associated with a degraded or absent blood clot in the socket, with or without halitosis, and without other potential causes of pain on the same side of the face (1). Its prevalence is reported to range from 1% to 5% (2), but can exceed 30% after the extraction of impacted mandibular third molars (3). Symptoms include severe, pulsating pain that may radiate to the ear and neck, along with oral malodor and poor taste. Clinical observations often reveal exposed bone due to the absence of a clot, which leads to about 45% of patients requiring additional dental visits for recovery (4). Risk factors include inadequate expertise of the surgeon, inadequate cleaning of the extraction socket intraoperatively, immunosuppression (5), difficulty of the procedure (6), the intake of contraceptives (7), smoking (8), and patient's age and gender (9). The primary cause is believed to be elevated fibrinolytic activity, resulting in premature clot disintegration, triggered by kinin release from tissue trauma and nerve exposure within the socket (10). The pain often does not respond to conventional analgesic agents prescribed postoperatively, making the treatment of dry sockets a highly debated topic.

To date, symptomatic therapy is most commonly performed for pain relief. While in certain situations, the systemic administration of analgesics or antibiotics may also be required, the application of intra-alveolar dressings is a suggested routine intervention to address this pain (11). Alvogyl® is among the most commonly used dressings for the treatment of dry socket, quickly relieving pain, and has a sustained analgesic effect during the treatment course. It contains eugenol, which has analgesic and antimicrobial properties; butamben, an anesthetic; and iodoform, with antimicrobial effects (12). Typically, 0.2 g of the paste is applied to the extraction socket, where it is absorbed within 24 hours. However, challenges such as high cost and limited availability in certain markets, such as Iran, underscore the need for alternative treatment options.

Thus, the gap in accessible and cost-effective interventions for managing dry socket has prompted the exploration of novel therapeutic approaches. This study aimed to experimentally prepare a topical dressing for dry socket treatment and compare its efficacy with that of Alvogyl®.

## Material and Methods

This double-blind, randomized, parallel-armed clinical trial was conducted on patients who presented to the Oral and Maxillofacial Surgery Department of Mashhad Dental School between 2020 and 2021. The experimental dressing formulation and other laboratory procedures, including herbal extract preparation, were carried out at the School of Pharmacy and Dental Materials Research Laboratory at Mashhad University of Medical Sciences in Mashhad, Iran, during the period from September 2020 to November 2020. The study was carried out in two phases: (I) preparation of the experimental dressing and (II) assessment of its clinical efficacy.

- (I) Formulation and preparation of the topical dressing

First, the effects of the constituents of Alvogyl® (Septodont, France) were evaluated by the faculty members of the pharmacology group of Mashhad University of Medical Sciences, and a formulation of the experimental dressing was designed. Oral triamcinolone (RAHA Pharmaceutical Co., Iran), ground Dianthus caryophyllus, eugenol (Morvabon, Iran), honey, and Iris germanica were used in its composition. The plant materials were collected in the spring from Mashhad (Northeast Iran) and were subsequently identified at the herbarium of the Faculty of Pharmacy at Mashhad University of Medical Sciences. Eugenol provides immediate analgesic effects, but its efficacy is short-lived as it is washed away by saliva. The ground Dianthus caryophyllus was used because of proliferative, antimicrobial and also analgesic properties found for Dianthus species (13). Iris germanica has also been used since it enhances healing, as it is a traditional healer in many cultures (14). Honey was added as a natural sweetener and an effective disinfecting agent (15-17). Oral triamcinolone paste was used to benefit from its paste-like consistency and to decrease inflammation and preserve the therapeutic agents in the extraction socket.

To prepare 100 g of the experimental dressing, 80 g of triamcinolone, 5 mL of eugenol, 10 g of ground Dianthus caryophyllus, 5 g of honey, and 5 g of ground Iris germanica were used. The dressing was then packed in V-Packs and autoclave-sterilized. The homogeneity of the experimental dressing was then evaluated. It was homogeneous and smooth when applied to the skin. One month after packaging the dressing in 100 g packs, the dressing maintained a homogenous consistency, was monophasic, and exhibited neither discoloration nor bad odor, ensuring its optimal stability over time. To assess its allergenicity, the dressing was implanted subcutaneously on the back of a guinea pig, and the area was monitored for 24 hours. No allergic reactions were observed after 24 hours. Next, the dressing was applied to the arm skin of 5 patients with no history of allergies, and they were monitored for allergy signs and symptoms. Accordingly, the dressing was found to be nonallergenic and safe.

- (II) Assessment of clinical efficacy

After the initial examination and selection of eligible patients, they were briefed about the study and signed informed consent forms. The study was approved by the ethics committee of the university (IR.MUI.RESEARCH.REC.1398.216) and registered in the Iranian Registry of Clinical Trials (IRCT20200706048029N1) on August 24th, 2020.

Since no similar study was found in the literature for sample size calculation, a pilot study was carried out, and the sample size in each group (*n*=18) was calculated accordingly. Eligible patients were selected by targeted convenience sampling. The inclusion criteria were an age between 30 and 60 years, presence of dry socket, and signed informed consent forms. The exclusion criteria were pregnancy, history of allergy to foods or medications, diabetes mellitus, systemic diseases, use of medications interfering with wound healing (corticosteroids, anti-cancer medications), active infectious diseases (hepatitis, tuberculosis, AIDS), and not showing up for the follow-ups. The dry socket was diagnosed by an oral and maxillofacial surgeon (K.F.) based on the presence of severe pain around the extraction socket starting at 1 to 3 days after tooth extraction and observation of an extraction socket without a blood clot and with exposed bone (7). To randomize group assignment, 32 envelopes were prepared by a person: half marked 'Group 1' and half 'Group 2'. Then patients randomly selected an envelope, and another person helped them determine their group. In this double-blind study, the patients and the assessor (A.S.) were not aware of the treatment groups.

While application of dressings was carried out by the person who diagnosed the dry socket (K.F.), a visual analog scale (VAS) was used by another person (A.S.) to assess the level of pain experienced by patients at the study onset and after the application of dressings. The VAS score ranged from 0 to 10 (18): a score of 0 indicated no pain; 1-2, very mild pain; 3-4, mild pain; 5-6, moderate pain; 7-8, severe pain; 9, very severe pain; and 10, unbearable pain. After recording the baseline pain scores of the patients, the extraction sockets were gently irrigated with warm 20 mL saline. Following debridement, the experimental dressing was applied to the extraction sockets of patients in group 1, and Alvogyl® was applied to the sockets of patients in group 2. The sockets were then sutured using Mersilk 3.0 suture thread, with Figure of eight sutures applied to secure the dressing and prevent its immediate washout.

**Figure 1:**
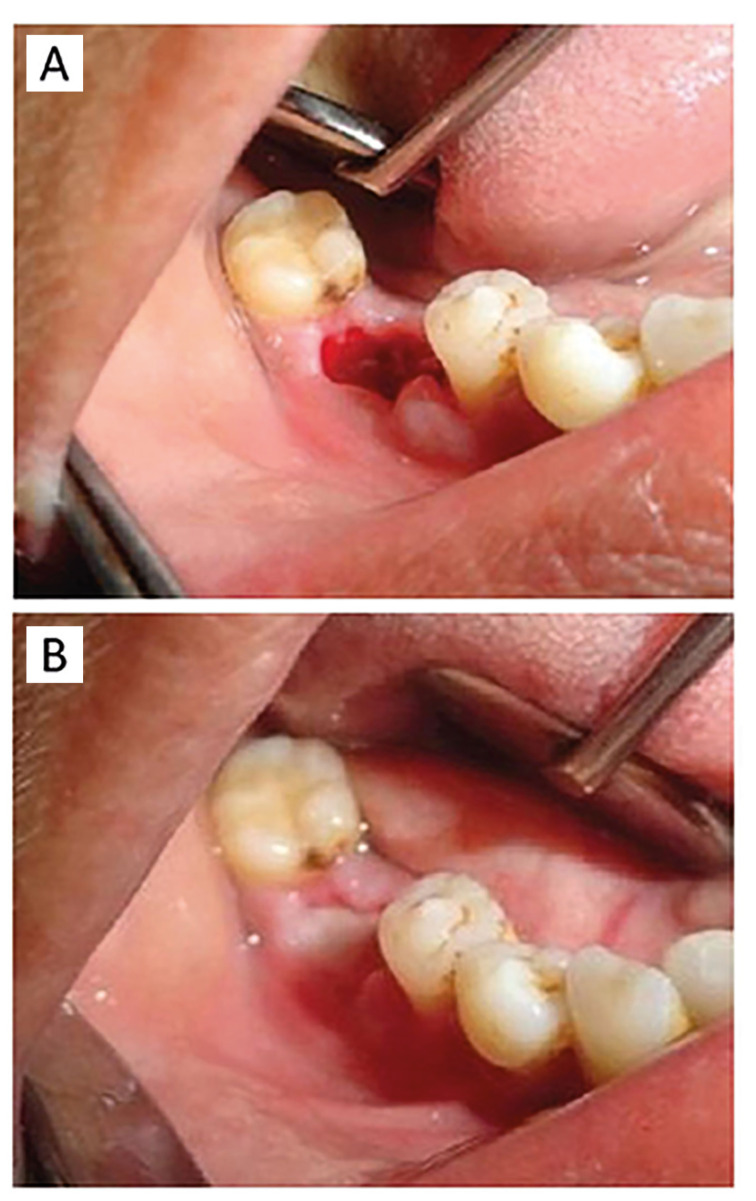
(A): extraction cavity in first visit. (B): cavity almost healed seven days after application.

The pain score of the patients was recorded immediately after the procedure (time zero) and at 30 and 60 minutes later. Afterward, patients were discharged and followed up after 24 hours to assess their pain level again. If patients reported severe pain and requested reapplication, the dressing would be reapplied, with each instance recorded on the checklist. Follow-ups were scheduled every 24 hours, continuing this cycle until complete pain resolution and healing signs were evident in the extraction socket, up to the 1-week follow-up (Fig. 1).

Finally, the frequency of dressing application for each patient was calculated. All patients were examined again after 1 week, and their pain score and number of analgesics taken during the week were recorded. For the purpose of standardization, all patients received ten 400 mg ibuprofen Tablets and were instructed to take one in case of pain. Additionally, demographic information (age, sex, tooth number) and phone number of patients were recorded on the checklist at study onset.

Complications including delayed healing, abscess, osteomyelitis, infection of pharyngeal spaces, and foreign body reaction were documented. Extraction sockets not healed by 10 days post-procedure were classified as experiencing delayed healing. These complications, alongside failure to attend follow-up appointments, constituted exclusion criteria.

- Statistical analysis

The data were analyzed using SPSS version 26 (Released 2018. IBM Corp., Armonk, NY, USA). The mean rank and difference with 95% confidence intervals were used to measure and compare the outcome data, with a 5% significance level. Upon applying the Shapiro-Wilk test, it was found that most of the quantitative variables, including pain scores, the number of analgesics taken, and the frequency of dressing applications, exhibited a non-normal distribution. The exception to this was age, which demonstrated a normal distribution. The two groups were compared in terms of age and sex using independent t-tests and chi-square tests, respectively. Pain scores at each time point, along with the number of analgesics consumed and the frequency of dressing applications, were evaluated between groups using the Mann-Whitney test. The Friedman test assessed changes in mean pain scores over time. For detailed analysis, pairwise comparisons of mean pain scores between two specific time points utilized Dunn’s multiple comparisons test.

## Results

A total of 36 patients participated in this study, including 20 females (55.5%) and 16 males (44.5%), with analyses completed on all participants without dropouts. The mean age of the patients in the experimental dressing group was 45.33±6.53 years (range 37 to 60 years), and that in the Alvogyl® group was 47.33±6.69 years (range 33 to 58 years), with no significant difference between the two groups (*p*=0.370). Additionally, there was no significant difference in sex distribution between groups, as there were 9 females (50%) in the experimental dressing group and 11 females (61.1%) in the Alvogyl® group. There were 9 (50%) males in the experimental dressing group and 7 (38.9%) in the Alvogyl® group (*p*=0.502).

- Comparison of the pain scores between the two groups at different time points

Table 1 presents the mean pain scores of the two groups at different time points. There was no significant difference in pain score between the two groups immediately after the intervention (*p*=0.888). The mean pain score in the experimental dressing group was significantly lower than that in the Alvogyl® group at 30 and 60 minutes after the intervention (*p*<0.001 for both). At 24 hours after the procedure, the mean pain score in the experimental dressing group was significantly greater than that in the Alvogyl® group (*p*=0.029). At 48 and 72 hours, the mean pain score was significantly greater in the experimental dressing group than in the Alvogyl® group (*p*=0.001 and *p*=0.017, respectively). The mean pain score in the experimental dressing group was slightly greater, but not significantly, than that in the Alvogyl® group at 96 hours and one week (*p*=0.584 and *p*=0.091, respectively).

- Within-group comparison of pain scores at different time points

**Figure 2:**
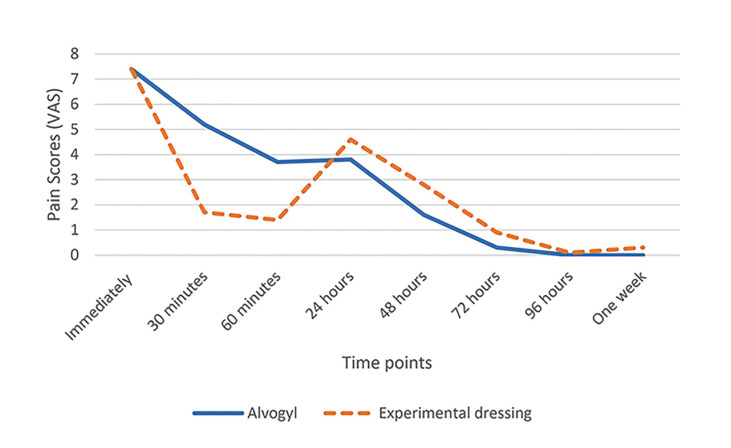
Mean pain scores at different time points within each group.

According to Table 2, in both groups, mean pain scores differed significantly over time (*p*<0.001). In the experimental dressing group, pain scores decreased significantly at all times except at 24 and 48 hours post-intervention compared to immediately after. At 30 minutes, pain was lower than at 24 hours (*p*=0.049) and higher than at 96 hours (*p*=0.017). Pain at 60 minutes was lower than that at 24 hours (*p*=0.010). Pain at 24 hours was higher than at 72 hours, 96 hours, and one week (*p*<0.001 for all). At 48 hours, pain was higher than at 96 hours and one week (*p*<0.001 for both). In the Alvogyl® group, pain scores at 48, 72, 96 hours, and one week were significantly lower than immediately and 30 minutes post-intervention (*p*<0.001). Pain scores at 72, 96 hours, and one week were lower than at 60 minutes and 24 hours (*p*=0.002 to *p*<0.001). The data are visualized clearly in Fig. 2.

Other comparisons were not significant for none of the groups (Table 3). The two groups were not significantly different regarding the number of analgesics taken (*p*=0.097) or the frequency of dressing application (*p*=0.839, Table 4).

- Harms and Complications

In both groups, no complications, including delayed recovery, abscess, osteomyelitis, space infection, or foreign body reactions, were found, and there were no cases of delayed socket healing.

## Discussion

Dry socket is among the most common complications of exodontia, often occurring 1 to 3 days after tooth extraction (1). The treatment of dry sockets remains a highly debated topic. To date, symptomatic therapy is most commonly performed for pain relief (19). Pain in dry sockets is caused by kinin release immediately after tissue trauma, exposure of nerve ends in the exposed bone to air, food, and drink, and an infectious process that activates pain mediators in the tissue (10). While in some certain situations, the systemic administration of analgesics or antibiotics may also be required (20), the application of intra-alveolar dressings is a suggested routine intervention to prevent simulation and pain (7). Alvogyl® is extensively used for the treatment of dry sockets, and it has been reported that it causes rapid pain relief in the course of treatment (11). The presence of eugenol in Alvogyl® is responsible for its fast analgesic efficacy (12). Alvogyl® is gradually dissolved in the extraction socket and inhibits pain receptors by inhibiting the release of pain mediators. Additionally, it serves as a protective barrier over the exposed bone (7).

Many other local interventions after tooth extraction have been recently assessed in the literature. Platelet-rich fibrin (PRF) advantages have been proved for the management of alveolar osteitis (21-23) and also as an alveolar ridge preserver (24). A 2022 study by Assari *et al*. (25) evaluated Alvogyl® and Cutanplast as intra-alveolar dressings for pain management and dry socket incidence after tooth extraction and found both comparable in postoperative pain management. A 2023 study by ALHarthi *et al*. (26) evaluated the efficacy of Alvogyl® with and without adjunct photobiomodulation therapy (PBMT) and found it efficient following mechanical curettage (MC). Other recent studies evaluated some herbal treatments. Alabdullah *et al*. study in 2023 (27) evaluated Nigella sativa oil compared to Eugenol. They found that Nigella sativa oil improved soft tissue healing and reduced inflammation severity in cases of dry socket. Khan *et al*. study at 2022 (28) evaluated Black Seed Oil compared to Alvogyl® and found the oil more efficacious dressing material for the management of dry sockets.

This study aimed to prepare an experimental dressing for dry socket treatment and compare its efficacy with that of Alvogyl®. The VAS is a standard tool for measuring pain intensity and is commonly used, with its validity and reliability previously confirmed in the literature (29). According to the comparative results between the two groups of this study, mean pain scores in the experimental group were significantly lower than those in the Alvogyl® group at 30 and 60 minutes, suggesting a faster analgesic effect of the experimental dressing. On the other hand, the mean pain score at 24, 48, and 72 hours in the experimental dressing group was significantly greater than that in the Alvogyl® group. The low efficacy of the experimental dressing at these time points may be because although the dressing caused immediate pain relief, it did not preserve its analgesic effect over time, probably due to its faster washout time compared to the Alvogyl®. Future studies should focus on using a medium to ensure longer substantivity of the dressing in the extraction socket. However, the results showed similar pain scores in the two groups immediately after the intervention, at 96 hours, and one week after, indicating comparable efficacy of the two dressings. Considering the duration of dry socket varies, typically lasting from 5 to 10 days (11), as it gets closer to the final days, probably no significant differences between the treatment dressings will be found.

Many other studies have assessed Alvogyl® efficacy, both alone and in comparison, with other treatment dressings. To the authors’ knowledge to this day, only one study with a new dressing could relieve pain better than Alvogyl®, reported by Khan *et al*. for Black Seed Oil (28). Supe *et al*. (5) reported that Alvogyl® provided faster pain relief than zinc oxide eugenol in dry socket treatment. Faizel *et al*. (12) found Alvogyl® more effective than zinc oxide eugenol and Neocone for primary pain relief. Eshghpour *et al*. (7) observed greater pain reduction in the Alvogyl® group than in the low-level laser group at 6 and 12 hours post-intervention. Ansari *et al*. (10) and Singh *et al*. (30) used organic honey for dry sockets and reported significant pain reduction after its application.

Considering the within-group results for Alvogyl® in our study, the application of Alvogyl® caused a significant reduction in pain scores at 48, 72, and 96 hours and one week after treatment compared to immediately after the intervention and at 30 minutes after the intervention. The mean pain scores at 72 and 96 hours and one week after the intervention were significantly lower than those at 60 minutes and 24 hours after the intervention and were approximately zero. Similarly, Eshghpour *et al*. (7) reported that Alvogyl® caused a significant reduction in pain on days 1 and 2 compared to day 0. They also observed an increase in pain score at 6 and 12 hours after the application of Alvogyl®, which indicated that Alvogyl® does not have short-term analgesic effect.

In this study, the two groups were standardized for age and sex due to the significant impact of these variables on dry socket development (12). The study's small sample size is a limitation. Future multicenter studies with larger sample sizes are recommended. Additionally, other variables such as surgery duration and procedure difficulty should be considered in future research for more accurate results.

## Conclusions

Considering the effectiveness and availability of the experimental dressing, and on the other hand, the high cost of Alvogyl®, this new treatment can be used as a more accessible alternative and also as an effective component in a new two active agent formulation.

## Figures and Tables

**Table 1:** Comparison of the mean pain scores of the two groups at different time points (n=18).

Time of pain assessment	Group	Mean ± SD*	Median (Interquartile range)	Minimum-Maximum	Mann-Whitney U test
Immediately	Experimental dressing	7.4 ± 1.2	7.0(2.3)	6-10	Z=0.16 *P*=0.888
	Alvogyl^®^	7.4 ± 1.4	7.0(1.5)	5-10	
30 minutes	Experimental dressing	1.7 ± 0.7	2.0(1.0)	1-3	Z=5.10 P<0.001
	Alvogyl^®^	5.2 ± 1.7	5.0(3.0)	3-8	
60 minutes	Experimental dressing	1.4 ± 0.5	1.0(1.0)	1-2	Z=5.01 P<0.001
	Alvogyl^®^	3.7 ± 1.0	3.5(2.0)	2-5	
24 hours	Experimental dressing	4.6 ± 1.1	5.0(1.3)	2-6	Z=2.25 *P*=0.029
	Alvogyl^®^	3.8 ± 1.0	4.0(1.3)	2-6	
48 hours	Experimental dressing	2.8 ± 1.1	3.0(1.3)	1-5	Z=3.24 *P*=0.001
	Alvogyl^®^	1.6 ± 0.9	1.5(1.0)	0-3	
72 hours	Experimental dressing	0.9 ± 0.9	1.0(1.3)	0-3	Z=2.64 *P*=0.017
	Alvogyl^®^	0.3 ± 0.6	0.0(0.3)	0-2	
96 hours	Experimental dressing	0.1 ± 0.3	0.0(0.0)	0-1	Z=1.43 *P*=0.584
	Alvogyl^®^	0.0 ± 0.0	0.0(0.0)	0-0	
One week	Experimental dressing	0.3 ± 0.5	0.0(1.0)	0-1	Z=2.65 *P*=0.091
	Alvogyl^®^	0.0 ± 0.0	0.0(0.0)	0-0	

*SD: Standard Deviation.

**Table 2:** Comparison of the mean pain scores at different time points within each group (n=18).

Group	Time of pain assessment	Mean ± standard deviation	Median (Interquartile range)	Mean rank*	Friedman test result
Experimental group	Immediately	7.4 ± 1.2	7.0(2.3)	7.94^a^	χ^2^=113.65 *P*<0.001
	30 minutes	1.7 ± 0.7	2.0(1.0)	4.42^bc^	
	60 minutes	1.4 ± 0.5	1.0(1.0)	4.06^bde^	
	24 hours	4.6 ± 1.1	5.0(1.3)	6.97^a^	
	48 hours	2.8 ± 1.1	3.0(1.3)	5.72^ace^	
	72 hours	0.9 ± 0.9	1.0(1.3)	3.22^bcd^	
	96 hours	0.1 ± 0.3	0.0(0.0)	1.61^df^	
	One week	0.3 ± 0.5	0.0(1.0)	2.06^bf^	
Alvogyl^®^	Immediately	7.4 ± 1.4	7.0(1.5)	7.97^a^	χ^2^=121.91 *P*<0.001
	30 minutes	5.2 ± 1.7	5.0(3.0)	6.83^a^	
	60 minutes	3.7 ± 1.0	3.5(2.0)	5.53^ac^	
	24 hours	3.8 ± 1.0	4.0(1.3)	5.61^ac^	
	48 hours	1.6 ± 0.9	1.5(1.0)	3.89^bc^	
	72 hours	0.3 ± 0.6	0.0(0.3)	2.28^b^	
	96 hours	0.0 ± 0.0	0.0(0.0)	1.94^b^	
	One week	0.0 ± 0.0	0.0(0.0)	1.94^b^	

*: Different lowercase letters in each group show significant difference between two time points.

**Table 3:** Pairwise comparisons of the mean pain scores at different time points within each group.

Time 1	Time 2	Experimental dressing	Alvogyl^®^
		*p-value*	*p-value*
Immediately	30 minutes	<0.001^*^	>0.99
	60 minutes	<0.001^*^	0.077
	24 hours	>0.99	0.107
	48 hours	0.182	<0.001^*^
	72 hours	<0.001^*^	<0.001^*^
	96 hours	<0.001^*^	<0.001^*^
	One week	<0.001^*^	<0.001^*^
30 minutes	60 minutes	>0.99	>0.99
	24 hours	0.049^*^	>0.99
	48 hours	>0.99	0.009^*^
	72 hours	>0.99	<0.001^*^
	96 hours	0.017	<0.001^*^
	One week	0.107	<0.001^*^
60 minutes	24 hours	0.010^*^	>0.99
	48 hours	>0.99	>0.99
	72 hours	>0.99	0.002^*^
	96 hours	0.077	<0.001^*^
	One week	0.401	<0.001^*^
24 hours	48 hours	>0.99	0.978
	72 hours	<0.001^*^	0.001^*^
	96 hours	<0.001^*^	<0.001^*^
	One week	<0.001^*^	<0.001^*^
48 hours	72 hours	0.062	>0.99
	96 hours	<0.001^*^	0.483
	One week	<0.001^*^	0.483
72 hours	96 hours	>0.99	>0.99
	One week	>0.99	>0.99
96 hours	One week	>0.99	>0.99

*Significant at *P*<0.05. Dunn's test.

**Table 4:** Comparison of the number of analgesics taken and the frequency of dressing applications in the two groups.

Group		Number	Mean ± SD*	Median (Interquartile range)	Mann-Whitney test result
Number of analgesics	Experimental dressing	18	2.7 ± 2.1	2.5(4.3)	Z=1.71 *p*=0.097
	Alvogyl	18	3.8 ± 1.5	3.0(2.0)	
Frequency of dressing application	Experimental dressing	18	2.9 ± 0.6	3.0(0.3)	Z=0.226 *p*=0.839
	Alvogyl	18	3.0 ± 0.8	3.0(2.0)	

*SD: Standard Deviation.
